# Post-translational modifications of EZH2 in cancer

**DOI:** 10.1186/s13578-020-00505-0

**Published:** 2020-12-11

**Authors:** Zhongwei Li, Minle Li, Diandian Wang, Pingfu Hou, Xintian Chen, Sufang Chu, Dafei Chai, Junnian Zheng, Jin Bai

**Affiliations:** 1 Cancer Institute, Xuzhou Medical University, 84 West Huaihai Road, Xuzhou, 221002 Jiangsu Province China; 2 Center of Clinical Oncology, Affiliated Hospital of Xuzhou Medical University, Xuzhou, 221002 Jiangsu Province China

**Keywords:** EZH2, Post-translational modification, Crosstalk, Cancer therapy

## Abstract

Enhancer of zeste homolog 2 (EZH2), as a main component of Polycomb Repressive Complex 2, catalyzes histone H3K27me3 to silence its target gene expression. EZH2 upregulation results in cancer development and poor prognosis of cancer patients. Post-translational modifications (PTMs) are important biological events in cancer progression. PTMs regulate protein conformation and diversity functions. Recently, mounting studies have demonstrated that EZH2 stability, histone methyltransferase activity, localization, and binding partners can be regulated by PTMs, including phosphorylation, *O*-GlcNAcylation, acetylation, methylation and ubiquitination. However, the detailed molecular mechanisms of the EZH2-PTMs and whether other types of PTMs occur in EZH2 remain largely unclear. This review presents an overview of different roles of EZH2 modification and EZH2-PTMs crosstalk during tumorigenesis and cancer metastasis. We also discussed the therapeutic potential of targeting EZH2 modifications for cancer therapy.

## Introduction

Dynamic regulation of histone modifications has a critical role in the modulation of gene expression [1]. Recent studies on cancer progression have observed aberrant expression of chromatin regulators that modify histones [2–7]. Previous studies have demonstrated the correlation between the chromatin modifier Enhancer of Zeste Homolog 2 (EZH2) and cancer tumorigenesis and metastasis [8–10]. EZH2, a key histone methyltransferase (HMTase), is the enzymatic subunit of Polycomb Repressive Complex 2 (PRC2), which catalyzes the trimethylation of lysine 27 of histone H3 (H3K27me3) leading to transcriptional silencing [11–14]. Since its discovery in 1996 [15, 16], only now has EZH2 been thought as an important histone methyltransferase in cancer progression [4, 17].

EZH2 reportedly promotes cancer development and metastasis [9, 17, 18]. EZH2 can regulate many cellular processes, such as migration, cell cycle, proliferation, DNA repair, apoptosis, and senescence, to facilitate cell survival or promote the malignant transformation of cells [8, 19–27]. For instance, EZH2 can promote the invasion and metastasis by suppressing E-cadherin transcriptional expression [28, 29]; EZH2 can also increase tumorigenesis by silencing tumor suppressors [9, 20, 25].

The functional regulation of EZH2 is important because of its several crucial roles in cancer progression. Many types of mechanisms regulate EZH2 functions, in particular with its expression, stability, and enzymatic activity [29–31]. For example, SOX4, Rb (retinoblastoma), and miR-101 can regulate EZH2 expression at the transcriptional or post-transcriptional level [32–34]. Evidence also indicates that the post-translational modifications (PTMs) of EZH2 are crucial for its protein stability, enzymatic activity, and its function in cancer development [18]. Among these diverse mechanisms, PTMs of EZH2 represent the most extensive and complicated types. The amino acids sequence of EZH2 protein makes it suitable for covalent modifications, including phosphorylation, acetylation, *O*-GlcNAcylation, methylation, ubiquitination, and sumoylation.

Recently, many reports have concentrated on the role of EZH2 modifications involved in phosphorylation, acetylation, methylation, ubiquitylation, *O*-GlcNAcylation, and sumoylation [31, 34–39]. Nevertheless, the amount of EZH2-PTMs studies is largely limited, and the mechanism by which different PTMs interplay in EZH2 remains unclear. Further studies in this aspect are still needed, and an overview of EZH2 PTMs in cancer development might help researchers fill in this research gap.

**Fig. 1:**
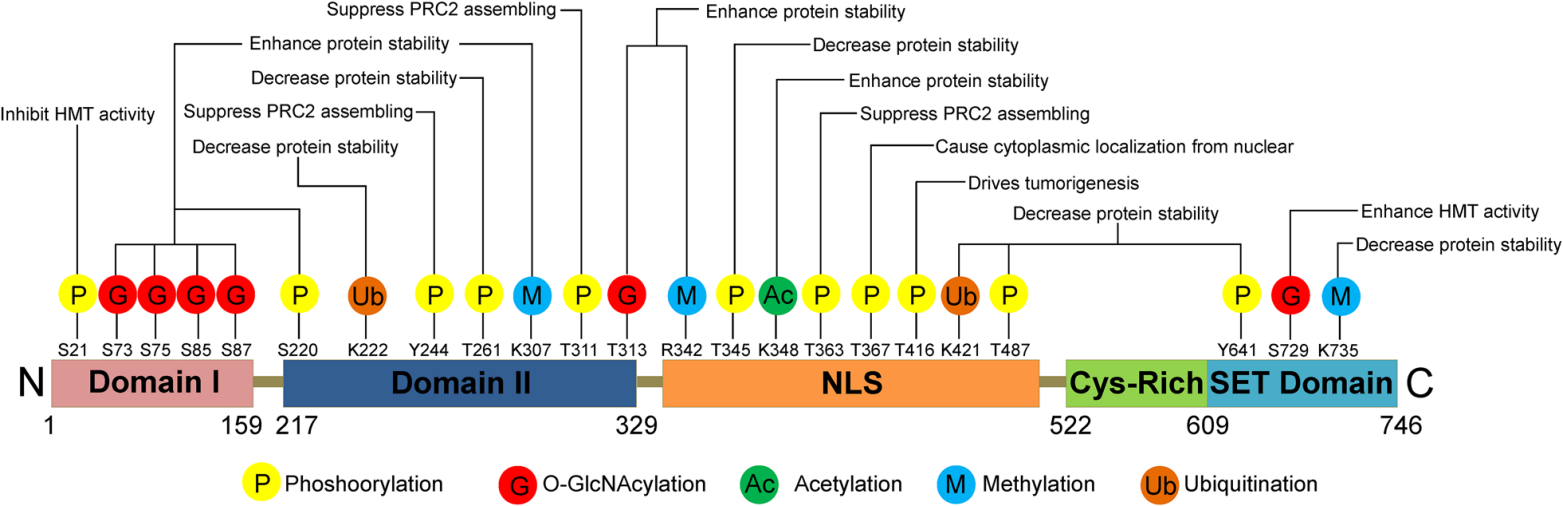
Overview of EZH2 PTMs. The major sites for EZH2 modifications (phosphorylation, *O*-GlcNAcylation, acetylation, methylation, ubiquitination) are plotted. Different colors are used to differentiate distinct modification types. Representative functions of these modifications are indicated

In the current review, we show an overview of the molecular mechanisms and biological functions of EZH2 modifications in cancer progression (Fig. 1 and Table 1). First, we introduced several typical examples of every kind of EZH2-PTMs. Then, the crosstalks or interplays between EZH2 PTMs were emphasized. Finally, the therapeutic potential of targeting EZH2 modifications also have been discussed.

**Table 1:** Functions of EZH2-PTMs

Type of PTMs	Site	Catalyzing enzyme	Biological functions	Cancer connection	Refs
Phosphorylation	S21	AKT	Promote tumorigenesis	Breast cancer, prostate cancer, GBM	[31, 33, 42]
	Y244	JAK3	Promote cells proliferation	NKTL	[45]
	S363	GSK3β	Decrease cells proliferation	Breast cancer	[46]
	T350 in human T345 in mouse	CDK1	Inhibit tumorigenesis and metastasis	Breast cancer, lung cancer,	[37, 74]
	T492 in human T487 in mouse	CDK1	Inhibit tumorigenesis and metastasis	Breast cancer, lung cancer,	[37, 74]
	Y646 in human Y641in mouse	JAK2	Inhibit tumorigenesis	Lymphoma	[48]
	T261	CDK5	Inhibit metastasis	Pancreatic cancer	[50]
	S220	MELK	Promote cells proliferation	NKTL	[51]
	T367	p38	Promote metastasis	Breast cancer	[52]
	T416	CDK2	Promote tumorigenesis	Breast cancer	[53, 54]
	T311	AMPK	Inhibit tumorigenesis	Breast cancer, ovarian cancer	[55]
*O*-GlcNAcylation	S73, S84, S87, T313 and S729	OGT	Regulates the stability and enzymatic activity of EZH2	Breast cancer	[36, 59]
Acetylation	K348	PCAF	Promote cell migration and invasion	Lung cancer	[37]
Methylation	K307	SMYD2	Promote tumorigenesis and metastasis	Breast cancer	[39]
	K735	SETD2	Inhibit metastasis	Prostate cancer	[73]
	K342	PRMT1	Promote metastasis	Breast cancer	[74, 75]
Ubiquitination	–	Praja1	Inhibit metastasis	Breast cancer	[38]
	K421	Smurf2	Inhibit metastasis	Prostate cancer	[85]
	-	β-TrCP	Inhibit tumorigenesis	Lymphoma	[48]
	–	TRAF6	Inhibit tumorigenesis and metastasis	Prostate cancer, breast cancer	[74, 84]
	–	FBW7	Inhibit cell migration and invasion	Pancreatic cancer	[50]
	–	CHIP	Inhibit tumorigenesis	Head and neck cancer	[83]
Deubiquitination	–	ZRANB1	Promote tumorigenesis and metastasis	Breast cancer	[99]
	–	USP7	Promote tumorigenesis and metastasis	Colon cancer prostate cancer	[101, 102]
	–	USP1	Promote tumorigenesis	Glioma	[103]
	K222	USP36	Promote cells proliferation	NKTL	[51]

### Phosphorylation of EZH2 in cancer development

Phosphorylation, as the best studied PTM, is an essential regulatory mechanism in several proteins [40]. Phosphorylation, which usually occurs on serine (S), threonine (T), and tyrosine (T) residues of substrates, induces a conformational change in many proteins, causing them to be activated or suppressed and consequently creating different biological functions [41]. On the EZH2 protein, numerous serine (S)/threonine (T) sites can be phosphorylated. This present review summarizes several main phosphorylation sites of EZH2 according to previous studies and related influence to their functions in cancer progression.

Phosphorylation is the earliest modification type identified on EZH2. As early as 2005, Cha et al. [31] showed phosphorylation of EZH2 at S21 (pS21-EZH2) by PI3K/AKT signaling in breast cancer cells. AKT-mediated pS21-EZH2 inhibits its methyltransferase activity by attenuating EZH2 associated with histone H3, which attenuates H3K27me3 level, increases EZH2 target genes expression, and facilitates breast cancer tumorigenesis. Although AKT-mediated-EZH2-S21 phosphorylation reduces its affinity toward histone H3, it does not change its subcellular localization or its interaction with Polycomb group protein SUZ12 and EED subunits. Cha et al. reported that the phosphorylated-EZH2 complex may promote oncogenesis by targeting other crucial nonhistone substrates in various cancers.

Instead of transcriptional repression EZH2 target gene expression, pS21-EZH2 serves as a transcriptional co-activator in castration-resistant prostate cancer through PI3K/AKT signaling [42]. They found that either knockdown of PRC2 components EED or SUZ12 or decreasing H3K27me3 exerts no functions on EZH2-activated target genes. It means that EZH2 can activate gene expression and oncogenesis without being dependent on its methyltransferase activity. Moreover, Bredfeldt et al. [43] showed that the membrane-activated estrogen receptor (ER) signaling pathway by diethylstilbestrol results in PI3K/AKT-mediated pS21-EZH2 increasing and decreasing H3K27me3 level in breast cancer MCF7 cells. This finding suggests that rapid ER signaling provides a direct linkage between xenoestrogen-induced nuclear hormone receptor signaling and modulation of epigenetic machinery during tissue development. Furthermore, Kim et al. [33] found that AKT-induced pS21-EZH2 elevates EZH2-mediated STAT3 methylation by increasing EZH2-STAT3 interaction in glioblastoma multiforme (GBM) stem-like cells. This report suggests that the AKT-pS21-EZH2-STAT3 signal pathway may have a great effect on regulating GBM tumorigenesis. Another study demonstrated that arsenic (As3+) stimulates AKT-mediated EZH2-S21 phosphorylation by regulating the JNK-STAT3-AKT signaling pathway [44]. This study discovered a new molecular mechanism about As3+ or other metal carcinogens induce tumorigenesis. Interestingly, arsenic-induced pS21-EZH2 is mainly cytoplasmic localization. This is different from the classical concept that EZH2 is mostly located in the nucleus. Reports on AKT-mediated pS21-EZH2 support the presumption that pS21-EZH2 mediated by AKT results in EZH2 promoting oncogenesis by several novel functions, which is independent on PRC2-mediated target gene transcriptional silencing.

Moreover, another study demonstrated that JAK3-mediated EZH2 tyrosine (Y) Y244 phosphorylation, which suppresses PRC2 complex formation, resulting in EZH2 oncogenic function independent of its HMTase activity in natural killer/T-cell lymphoma (NKTL) [45]. This report also showed a novel model of EZH2 oncogenic effect through JAK3-mediated Y244-EZH2 phosphorylation, which leads to EZH2 function from a gene repressor to a gene activator in NKTL cells [45]. Similarly, Hung and his colleagues discovered that GSK3β phosphorylating S363-EZH2 reduces H3K27me3 and attenuates breast cancer oncogenic function [46].

Chen et al. [35] reported that the phosphorylation of T350 in human (T345 in mouse) EZH2 (pT350-EZH2 or pT345-EZH2) is catalyzed by CDK1 or CDK2 in prostate cancer cells. They found that pT350-EZH2 is necessary for EZH2-mediated H3K27me3 modifications. They showed that pT350-EZH2 strengthens EZH2 target gene transcriptional silencing without affecting its histone methyltransferase activity or PRC2 complex aggregation. They also disclosed that pT350-EZH2 can elevate EZH2-mediated cell proliferation and migration. Moreover, another study revealed that CDK1 catylazes EZH2-T487 residue phosphorylation in mouse EZH2 (the counterpart of pT492-EZH2 in human) [47]. Although T487 and T345 are phosphorylated by CDK1, researchers found that the function of pT487-EZH2 is completely different from that of pT345-EZH2. They found that pT487-EZH2 inhibits H3K27me3 by decreasing EZH2 methyltransferase activity and attenuating EZH2 associated with other PRC2 subunits, thereby derepressing EZH2 target gene transcriptional silencing and inhibiting cells invasion and metastasis in breast cancer cells and human mesenchymal stem cells.

However, a report that also focused on CDK1-mediated EZH2 phosphorylation indicated that pT345-EZH2 and pT487-EZH2 are not indispensable for H3K27me3 formation [30]. They showed that pT345-EZH2 and pT487-EZH2 have no effect on EZH2 methyltransferase activity and can't repress its binding with other PRC2 components. They found that pT345-EZH2 and pT487-EZH2 facilitate EZH2 ubiquitination and hence its degradation by the proteasome pathway in human cervical cancer cells [30]. Our recent study has also confirmed that CDK1-mediated pT345-EZH2 and pT487-EZH2 facilitate EZH2 ubiquitination and subsequent degradation in breast cancer [28]. We indicated that the phosphorylation of T345-EZH2 and T487-EZH2 attenuates oncogenesis and metastasis in breast carcinoma by decreasing EZH2 stability. It’s very interesting that these mentioned studies showed different biological functions of CDK1-mediated phosphorylation of EZH2-T345 and EZH2-T487 sites. It suggest that CDK1-mediated EZH2 phosphorylation may have different functions in different knids of cell lines.

A study demonstrated that the phosphorylation of EZH2 at Y646 residue in human (Y641 in mouse) by JAK2 promotes the β-TrCP-mediated EZH2 degradation and consequent regulation of H3K27me3 [48]. They also found that JAK2-mediated pY641-EZH2 suppresses lymphoma pathogenesis. Besides, EZH2-Y646 single site mutation have been reported in cutaneous melanoma, follicular lymphoma, diffuse large B-cell lymphomas [48, 49]. For instance, about 28% of EZH2-Y646-mutation has been found in follicular lymphomas, which increases lymphoma cells H3K27me3 level and lymphoma pathogenesis [49]. Another recent study has reported that CDK5-mediated T261-EZH2 phosphorylation facilitates FBW7-mediated EZH2 ubiquitination and proteasome degradation in pancreatic cancer cells [50]. While, Li et al. [51] disclosed that MELK-mediated EZH2-S220 phoshoorylation attenulates EZH2 K222 ubiquitination in NKTL cells.

Talha et al. [52] revealed that p38 phosphorylated EZH2 at T367 site facilitating its cytoplasmic localization and interacting with vinculin and other cytoskeletal regulators of cell migration and invasion. Their study also revealed that pT367-EZH2 is essential for EZH2 cytoplasmic localization in breast cancer patients. This finding suggests that EZH2 can promote breast cancer metastasis through novel functions in cytoplasm. Interestingly, Hung et al. found that CDK2 phosphorylates EZH2 T416 sitee enhancing triple-negative breast cancer (TNBC) cell motility in vivo and in vitro. They also showed that CDK2-mediated pT416-EZH2 drives tumorigenesis [53, 54].

In 2018, Li et al. [55] demonstrated that AMPK phosphorylates EZH2 at T311 residue to inhibit EZH2 binding with SUZ12, thereby attenuating the PRC2-dependent methylation of H3K27 and enhancing PRC2 target genes translation in ovarian and breast cancers. Their data also showed that AMPK-mediated pT311-EZH2 represses the cells proliferation in ovarian and breast cancers. This report suggests that energy stresses such as glucose deprivation or glycolysis blockade activates AMPK kinase and subsequently relieves EZH2-mediated target gene silencing, which inhibits cancer cell proliferation. This is an example of AMPK as a critical pivot to connect cellular energy state and pathological development through the regulation of EZH2 phosphorylation in ovarian cancer and breast cancer cells.

In sum, phosphorylation of EZH2 is important for oncogenesis and metastasis in numerous cancer types. When phosphorylation of EZH2 occurs and what effects phosphorylation has on EZH2 are very changeable in different kinds of pathological conditions or different kinds of cancers.

### GlcNAc, acetylation, and methylation of EZH2 in cancer progression

Protein glycosylation with β-*N*-acetyl-d-glucosamine (*O*-GlcNAcylation, GlcNAc) is a reversible and dynamic PTM process ubiquitous in thousands of protein substrates [56, 57]. *O*-linked *N*-acetylglucosamine transferase (OGT) is the only known enzyme that catalyzes the *O*-GlcNAcylation of substrates at the side chain hydroxyl group of S or T residue [58]. Professor Wong's team first provided convincing evidence on OGT-mediated EZH2 *O*-GlcNAcylation at S75 in breast cancer [36]. This report also found that OGT-mediated *O*-GlcNAcylation at S75 stabilizes EZH2 and subsequently facilitates the formation of H3K27me3 on PRC2 target genes. They confirmed that the OGT-EZH2 axis inhibits tumor suppression by repressing the expression of several key tumor suppression genes in breast carcinoma. Interestingly, Wong et al. [59] also identified *O*-GlcNAcylation at the S73, S84, S87, T313, and S729 residues of EZH2 in 2018. Their data showed that these identified GlcNAcylation sites of EZH2 have different biological functions. On the one hand, they showed that single mutants of EZH2 at S73, S84, S87, T313, and S729 residues reduce protein stability compared with the wild-type EZH2 and that the *O*-GlcNAcylation of EZH2 on the five sites does not affect its association with the PRC2 complex subunits (SUZ12, EED, and RBBP4/7). Conversely, they revealed that *O*-GlcNAcylation at S729 in the EZH2 SET domain strengthens the methyltransferase activity and promotes the formation of H3K27me2/3 on EZH2 target gene. It means that OGT-mediated EZH2-GlcNAcylation have several different functions in breast cancer progression. Acetylation is a reversible and important PTM that regulates a series of cellular processes, including proliferation, apoptosis, migration, and metabolism, in cancer cells; it is achieved through the modulation of core histones or non-histone proteins by histone acetyltransferases (HATs) or histone deacetylases (HDACs) [60–66]. Recently, Wan et al. [37] have elucidated that EZH2-K348 residue is acetylated by acetyltransferase P300/CBP-associated factor (PCAF) and is deacetylated by deacetylase SIRT1 in lung cancer cells. They discovered that PCAF-mediated EZH2-K348 increases EZH2 stability, facilitates its capacity in repression of its target genes, and strengthens lung cancer cell migration and invasion. Just as this study showed, all the study of EZH2-K348 acetylation functions is dependent on in vitro lung cancer cell lines experiments. Whether EZH2-K348 acetylation has the similar functions in mouse model in vivo experiment, which is still unknown.

Similar to acetylation, methylation is also a reversible and critical epigenetic marker at the lysine or arginine of histones or non-histone proteins [67–72]. In 2019, Zeng et al. [39] found that SET and MYND domain containing 2 (SMYD2)-mediated EZH2 di-methylation at lysine 307 (K307) enhances its stability, which can be demethylated by the histone H3K4 demethylase lysine-specific demethylase 1 (LSD1) in breast cancer. This study indicated that EZH2-K307 di-methylation promotes the proliferation and invasion of breast cancer cells through facilitating the recruitment of EZH2 to chromatin and the subsequent transcriptional repression of EZH2 target genes.

In 2020, Yuan et al. [73] reported that SETD2 methylates EZH2 at K735 promoting EZH2 degradation and impeding prostate cancer metastasis. Mechanically, they demonstrated that SETD2-mediated EZH2-K735me1 facilitates E3 ligase Smurf binding with EZH2 and degradation of EZH2. In addition, our newest study found that PRMT1-mediated R342-EZH2 asymmetric di-methylation (ADMA) strengthens EZH2 stability and promotes breast cancer metastasis. We showed that R342-EZH2 methylation inhibits TRAF6-mediated EZH2 ubiquitination [74]. We also demonstrated that tumor-associated maceophages (TAMs) facilitate PRMT1-mediated R342-EZH2 ADMA methylation by secreting IL-6, which strengthens EZH2 protein stability and enhances breast cancer cells motility [75].

### Ubiquitination, sumoylation, and deubiquitination of EZH2 in tumorigenesis and cancer metastasis

Ubiquitin (Ub), including 76 amino acid residues, is an evolutionarily highly conserved protein dedicated to tagging target proteins for post-translational degradation. Ubiquitination is a well-known PTM process that covalently adds Ub to the modified protein substrates and regulates their stability, biological functions, and localizations [76–79]. Ubiquitination is involved in multiple functions and diseases, especially in tumorigenesis and cancer metastasis [77, 78, 80–82]. It occurs by a well-organized cascade of enzymatic reactions dependent on three indispensable enzymes—an E1 ubiquitin activating enzyme, an E2 ubiquitin-conjugating enzyme, and an E3 ubiquitin-ligating enzyme. Up to now, several E3 ligases have been identified mediating the degradation of EZH2 through the ubiquitin–proteasome pathway; these ligases include Praja1, Smad ubiquitination regulatory factor-2 (Smurf2), β-TrCP (also called FBXW1), TRAF6, Casitas B-lineage lymphoma (c-Cbl), Fbw7 and CHIP (COOH terminus of Hsp70-interacting protein) [38, 48, 50, 83–89].

The first research of EZH2 ubiquitination was from the Aaron lab’s work in 2011 [88]. They found that Ub E3 ligase Praja1 mediates EZH2 protein degradation through the ubiquitination-proteasome pathway in MCF7 cells (breast cancer cell line). Aaron and his colleagues illustrated that Praja1 promotes EZH2 degradation through K48-linkage polyubiquitination and suppresses cells growth and migration in breast cancer [87]. They also showed that FOXP3 can promote the degradation of the EZH2 ubiquitination–proteasome pathway by accelerating the transcriptional expression of Praja1 directly. Recently, a report has confirmed that Praja1 degrades EZH2 during skeletal myogenesis [38]. This finding disclosed that Praja1-mediated EZH2 degradation is required for muscle satellite cells differentiation.

Aside from Praja1, other E3 ligases target EZH2. A study revealed that Smurf2 can interact with EZH2 and mediate EZH2 ubiquitination-proteasome degradation. This study also found that lysine 421 of EZH2 plays a key part in Smurf2-mediated EZH2 degradation [85]. A recent report has shown that YC-1 stimulates E3 ligase c-Cbl-mediated EZH2 ubiquitination and proteasomal degradation in breast cancer [86]. Mechanistically, YC-1 treatment promotes c-Cbl phosphorylation at T731 and T774, which results in c-Cbl-induced Src and ERK activation, leading to the formation of the c-Cbl-ERK-EZH2 complex and the consequent accumulation of EZH2 ubiquitination and proteasomal degradation. Furthermore, SCF E3 ubiquitin ligase β-TrCP reduces EZH2 stability and H3K27me3 occupation through mediating EZH2 ubiquitination-proteasome degradation in breast cancer cells [48]. Lu et al. [84] discovered that TRAF6 catalyzes the K63-linked polyubiquitination of EZH2, thus decreasing EZH2 and H3K27me3 levels in prostate cancer cells and prostate patients. Moreover, Jin et al. [50] revealed that FBW7 decreases EZH2 activity and attenuates the motility of pancreatic cancer cells by mediating the degradation of the EZH2 ubiquitin proteasome pathway. Moreover, researchers demonstrated that E3 ligase CHIP can mediate EZH2 ubiquitination degradation and subsequently derepress EZH2-silenced tumor suppressor genes by attenuating the H3K27me3 level in head and neck cancer cells [83].

Like ubiquitylation, there is also a ubiquitin-like proteins, small ubiquitin-like modifier (SUMO) [90]. The process is called sumoylation. Sumoylation is a highly conserved enzymatic cascade in whichSUMO proteins are conjugated to certain lysine residues via a similar mechanism to ubiquitination [91]. A study showed that sumoylation of EZH2 is associated with EZH2 activity in U2OS cell (osteosarcoma cell line) [34]. Nevertheless, they did not figure out the EZH2 specific sumoylation residue and the molecular mechanism of how EZH2 is involved in sumoylation.

Ubiquitination mediates targeted protein degradation, whereas deubiquitinases (DUBs, also called deubiquitylases) can remove ubiquitin molecules from ubiquitin-labeled proteins or from polyubiquitin chains to strengthen the stability of targeted proteins [92, 93]. DUBs play a critical part in cancer metastasis and tumorigenesis [94–98]. Zhang et al. [99] identified ZRANB1 (also named Trabid) as an EZH2 DUB. They demonstrated that ZRANB1 can bind, deubiquitinate, and stabilize EZH2, which enhances breast cancer tumorigenesis and metastasis. Ubiquitin-specific protease 7 (USP7), also known as herpesvirus-associated ubiquitin-specific protease, is a deubiquitinating enzyme that can deubiquitinate EZH2 [100–102]. Gagarina et al. [101] found that USP7 can stabilize EZH2 and facilitate EZH2-mediated H3K27me3 levels in HCT116 colon carcinoma cells. Moreover, another research illustrated that USP7 elevates EZH2 stability by mediating EZH2 deubiquitination in prostate cancer cells [102]. They also demonstrated that USP7-mediated EZH2-deubiquitination increases the cells ability of growth and motility and the tumorigenesis and metastatic invasive activity in vivo of prostate cancer cells. Besides, Ma and his colleagues found that Ubiquitin-specific protease 1 (USP1) directly interacts with and deubiquitinates EZH2. USP1-mediated stabilization of EZH2 enhances the accumulation of H3K27me3 and decreases its target gene expression, which drives the proliferation and tumorigenesis of glioma cells [103]. Recently, Chng et al. [51] demonstrated that Ubiquitin-specific protease 36 (USP36) deubiquitinates EZH2 at K222 site to strengthen EZH2 stability in NKTL. Taken together, these reports suggest that ZRANB1, USP7, USP1 and USP36 can promote cancer development via the stabilization of EZH2 by deubiquitination.

### Crosstalk between phosphorylation of EZH2 and ncRNAs

Recently, Xist et al. have confirmed that numerous long noncoding RNAs (lncRNAs), such as ANCR (also known as DANCR) and HOTAIR, interact with EZH2 [28, 104–106]. Most of those EZH2-binding lncRNAs play a key part in cancer development. Surprisingly, a report discovered that pT345-EZH2 plays an important part in lncRNA binding with EZH2 [107]. Because their study indicated that the EZH2-T345D mutant shows stronger ability of association with HOTAIR and Xist lncRNAs than the wild-type and T345A mutant version of EZH2. In addition, they identified a ncRNA-binding domain (ncRBD1) from R342 residue to T370 in full-length EZH2, containing the T345 phosphorylation site. Their study demonstrated that the phosphorylation of EZH2 influences lncRNAs-EZH2 interaction.

Our recent research has illustrated that ANCR, a type of lncRNAs, promotes EZH2-T345 phosphorylation by associating with EZH2 [28]. We disclosed that ANCR-EZH2 interaction enhances CDK1 binding with EZH2 and increases the amount of pT345-EZH2, which results in EZH2 degradation and subsequently suppressing the oncogenesis and distant metastasis in breast cancer. We speculate that ANCR-EZH2 association may change the conformation of EZH2, which probably facilitates the recognition and binding of CDK1 on EZH2 to phosphorylate its T345 residue.

Sun et al. found that circ-ADD3, as a circular RNA, inhibits hepatocellular carcinoma (HCC) metastasis through facilitating EZH2 degradation through CDK1-mediated EZH2 ubiquitination [108]. They confirmed that circ-ADD3 binding with EZH2 facilitates CDK1-mediated EZH2 phosphorylation on T345 and T487, which results in EZH2 ubiquitination degradation in HCC cells.

Taken together, these studies demonstrated that a crosstalk occurs between the phosphorylation of EZH2 and ncRNAs. We speculate that other underlying interactions occur between the phosphorylation of EZH2 and ncRNAs. Understanding the underlying functions and mechanisms of the crosstalk between the phosphorylation of EZH2 and ncRNAs will break a new path for further clarification of EZH2 functions in PRC2 complex-involved epigenetic regulation.

### Crosstalk between PTMs of EZH2

**Fig. 2:**
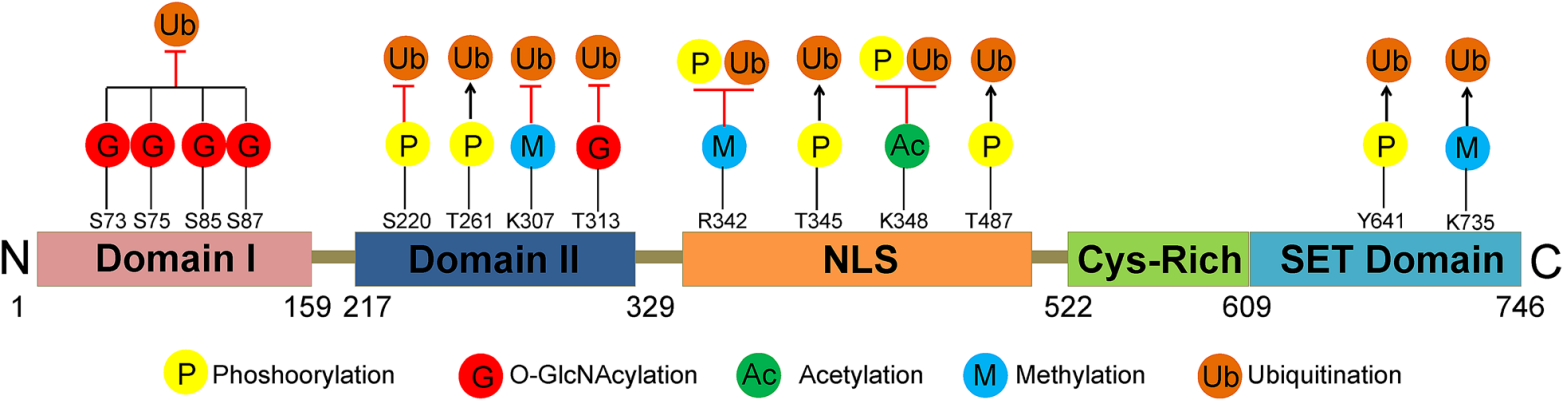
Crosstalk between EZH2 PTMs. There are widespread interplays between EZH2 modifications. These crosstalks can be divided into cooperative or antagonistic modification crosstalk. For instance, cooperative modification crosstalk (the case that T261 phosphorylation promotes EZH2 ubiquitination); antagonistic modification crosstalk (the case that K348 acetylation inhibits T345 and T487 phosphorylations and following EZH2 ubiquitination). For detailed discussion, see the context. Black arrows indicate positive effects (cooperative modification crosstalk); Red perpendicular bars indicate negative effects (antagonistic modification crosstalk)

As mentioned above, a series of residues in EZH2 can undergo various PTMs, thereby rising a question about the significance of a single-site modification in EZH2 function modulation. In fact, in most cases, dependence on a single residue PTM is hardly decisive. Comprehensive crosstalk occurs among PTMs of EZH2 (Fig. 2). According to the EZH2-PTM effect of promotion or inhibition to each other, we can classify them as cooperative modification crosstalk (one modification promotes or enhances the effect of another) vs. antagonistic modification crosstalk (one modification antagonizes the effect of another).

Most the cases for EZH2-PTMs crosstalk is that some amino acids in EZH2 can be modified, thereby affecting its ubiquitination. For instance, CDK1-mediated pT345-EZH2 and pT487-EZH2 facilitate EZH2 ubiquitination degradation in breast cancer cell, cervical cancer cell and lung cancer cell [28, 30, 108]; JAK2 phosphorylates Y641-EZH2, leading to E3 ligase β-TrCP-mediated EZH2 degradation in lymphoma cell [48]; and CDK5 phosphorylation of EZH2 at T261 residue results in the E3 ubiquitin ligase FBW7-mediated degradation of EZH2 in pancreatic cancer cell [50]. Silvia et al. [38] revealed that p38α promotes E3 ligase Praja1-mediated EZH2 degradation through the phosphorylation of T372-EZH2 (T367-EZH2 in mouse). SETD2-mediated mono-methylation of EZH2-K735 promotes EZH2 ubiquitination in prostate cancer [73]. All of these studies show a cooperative modification crosstalk of EZH2.

By contrast, many studies found an antagonistic modification crosstalk on EZH2 PTMs. OGT-mediated *O*-GlcNAcylation of EZH2 attenuates EZH2 ubiquitination in breast cancer cell [36, 59]; PCAF-mediated EZH2-K348 acetylation inhibits CDK1 catalyzing pT345-EZH2 and pT487-EZH2 and increases EZH2 stability in lung cancer [37]. Nuclear inhibitor of PP1 (NIPP1) dephosphorylates the CDK1-mediated pT345-EZH2 and pT487-EZH2 residues, thereby strengthening the stabilization of EZH2 [109]. MELK phosphorylating EZH2-S220 prevents EZH2-K222-mediated ubiquitination in NKTL cells [51]. Moreover, a recent report has revealed that SYDM2 catalyzes EZH2-K307 di-methylation attenuating EZH2-ubiquitination degradation in breast cancer [39]. In addition, our recently studies discovered that PRMT1-mediated EZH2-R342 methylation attenuates CDK1-mediated EZH2-T345 and EZH2-T487 phosphorylation, which strengthens EZH2 stability [74, 75].

In conclusion, the crosstalks of EZH2 PTMs are variable and complicated. In fact, the majority of those crosstalks have not been totally illustrated, suggesting that further investigations are needed to explore other crosstalks of EZH2-PTMs.

### Targeting the EZH2-PTM pathway for cancer therapy

**Fig. 3:**
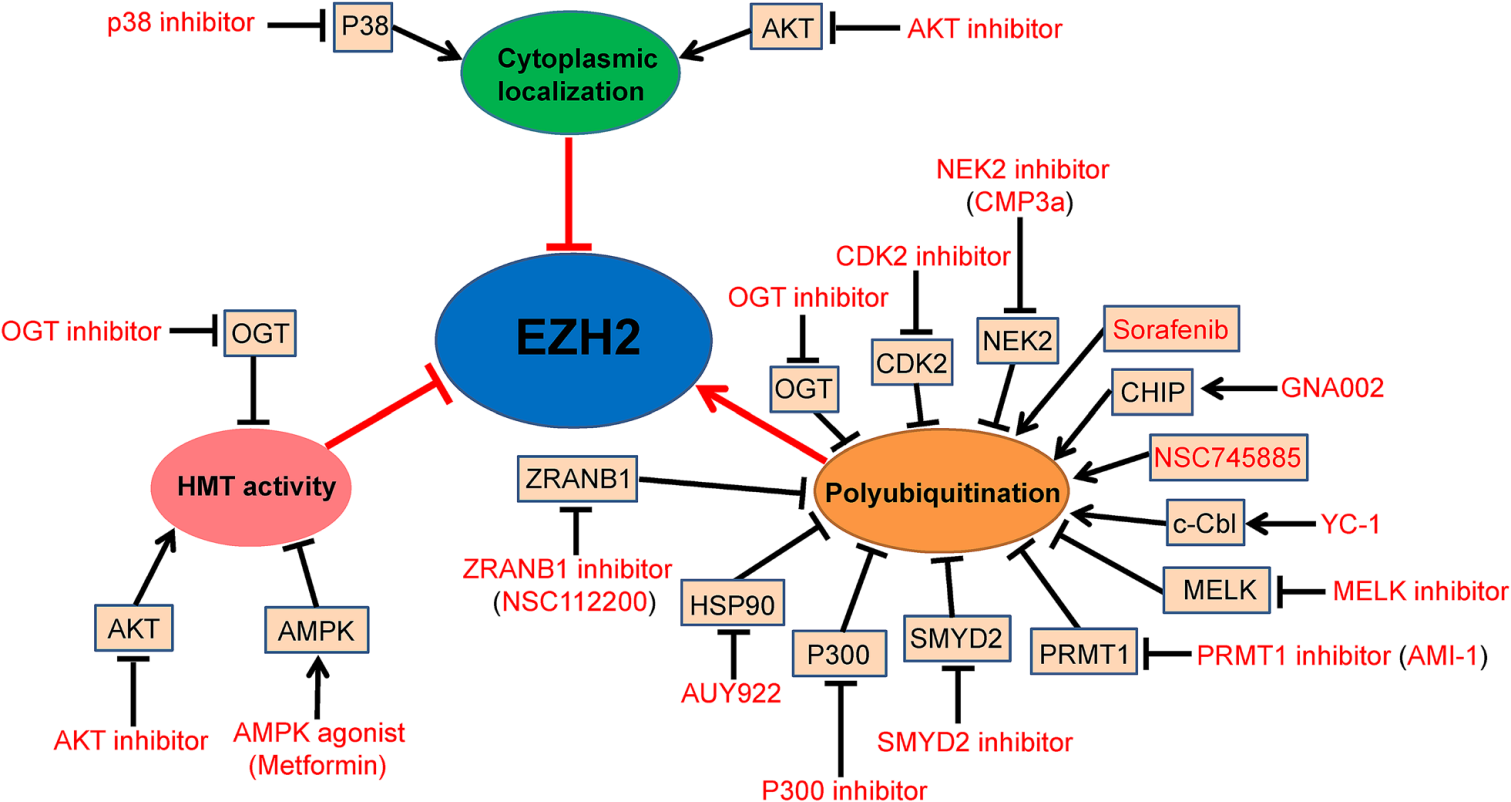
Targeting EZH2 modification for cancer therapy. Overview of the moleculars (black) and pharmacologic small-molecular compounds (red) regulations of EZH2. A lot of small-molecular compounds have been developed to target the major enzymes, which can regulate EZH2 HMT activity and EZH2 ubiquitination modifications. Arrows indicate positive effects, and perpendicular bars indicate negative effects

EZH2 is overexpressed in many kinds of cancers [9]. Inhibiting the biological function of EZH2 is a new focus in drug discovery [10, 103, 110]. The expression, stability, localization, and activity of EZH2 can be disrupted through different mechanisms in patients with cancer. As shown above, EZH2 PTMs can influence EZH2 functions in several aspects, making it an attractive therapeutic target to prevent EZH2 functions in cancer. Widespread studies have focused on targeting the modifications of EZH2 function (Fig. 3).

As we discussed, the PTM of the EZH2-ubiquitination pathway is an important negative regulator of EZH2. A series of small molecules have been shown to facilitate EZH2-ubiquitination degradation. YC-1(Lificiguat), 3-(5′-hydroxymethyl-2′-furyl)-1-benzylindazole, is an allosteric activator of soluble guanylyl cyclase. YC-1 can attenuate platelet accumulation and prevent vascular contraction [111]. Previous reports demonstrated the potent anticancer activity of YC-1 in many cancer cells [112–114]. A study reported that YC-1 decreases EZH2 expression and inhibits breast cancer cell proliferation via activation of its ubiquitination and proteasome degradation [86]. Mechanically, they elucidated that YC-1 facilitates E3-ligase c-Cbl phosphorylation at T731 and T774, leading to the activation of c-Cbl and complex formation with EZH2, and then EZH2 ubiquitination degradation.

Sorafenib, as a multikinase inhibitor, is a phase III clinical trial drug in advanced hepatocellular carcinoma patients [115, 116]. A recent research has disclosed that sorafenib can prevent EZH2 expression by accelerating its ubiquitination-proteasome degradation in hepatoma cells [117]. Moreover, NSC745885, as a small molecular, is derived from natural anthraquinone emodin, which can downregulate EZH2 via proteasome-mediated degradation [22]. NSC745885 shows potent selective toxicity against various cancer cell lines but not normal cells. The researchers also demonstrated that NSC745885 suppresses bladder tumorigenesis and downregulates EZH2 in vivo. It means that NSC745885 has potent anti-cancer effects through facilitating EZH2 ubiquitination-proteasome degradation. Therefore, NSC745885 is a hopeful therapeutic target in bladder cancer patients.

Luminespib (AUY-922, NVP-AUY922), as a highly potent HSP90 inhibitor [118], can destabilize T-cell EZH2 protein by inhibiting HSP90 function [119]. A study found that NIMA-related kinase 2 (NEK2) strengthens EZH2 protein stability to promote GBM tumorigenesis [120]. Subsequently, the researchers designed a highly specific NEK2 inhibitor, CMP3a, which can promote EZH2 ubiquitination degradation and inhibit GBM tumor growth. Interestingly, small-molecule GNA002, as a gambogenic acid (GNA) derivative, was identified as a potentially stronger EZH2 inhibitor [83]. Researchers indicated that GNA002 can specific bind to Cys668 within the EZH2-SET domain, stimulating EZH2 degradation through the COOH terminus of CHIP-mediated ubiquitination. Their discovery indicated that GNA002-mediated ubiquitination-degradation of EZH2 may become a promising treatment strategy for head and neck carcinoma patients. CDK2 inhibitor dinaciclib renders the high-grade serous ovarian carcinoma (HGSOC) cells sensitive to hormonal therapy (tamoxifen) by increasing ERα expression [121]. It means that CDK2 inhibitor combining with tamoxifen is a promising therapeutic strategy of HGSOC in the future. In addition, our recently study showed that PRMT1 specific inhibitor AMI-1 inhibits breast cancer cell invasion and migration through facilitate EZH2 degradation [74]. Our study suggest that PRMT1 inhibitor is a promising clinical drug to decreasing breast cancer patients metastasis.

Aside from these small molecules, certain inhibitors may also be used as potential anti-cancer strategies to inhibit EZH2 HMTase activity or protein stability. For instance, AKT-mediated pS21-EZH2 can promote breast cancer tumorigenesis [31, 42]. Thus, AKT specific inhibitors are possible targets for many cancers. OGT catalyzing *O*-GlcNAcylation of EZH2-S75 strengthens EZH2 stability, which means that OGT inhibitors (such as OSMI-1) are possible therapeutic targets for breast cancers [36, 58]. Moreover, PCAF acetylates EZH2 at the K348 site promoting lung cancer tumorigenesis via stabilizing EZH2 [37]. PCAF inhibitors may develop into an anti-cancer target. In addition, p38 catalyzing EZH2 phosphorylation at T367 residue elevates its localized to cytoplasm and promotes breast cancer cells distant metastasis [52]. We speculated that targeting p38-mediated pT367-EZH2 by p38 inhibitor may develop into a potential therapeutic strategy to prevent breast cancer distant metastasis.

Wei and his colleagues found that the AMPK-mediated phosphorylation of EZH2 at T311 decreases its methyltransferase activity to relieve the EZH2-dependent epigenetic silencing of its target genes and subsequently suppresses ovarian cancer tumorigenesis [55]. This study also elucidated that AMPK-mediated pT311-EZH2 correlates with high survival rate in patients with ovarian cancer. This finding clearly suggests that AMPK agonists may become promising sensitizers of EZH2-targeting drugs in cancer patients [55]. Among currently available AMPK agonists, metformin, as a first-line drug for type II diabetes mellitus patients, has already been widely used in pre-clinical studies to investigate the effect of energy restriction on cancer cells. Interestingly, most studies support an anti-cancer function of metformin, which is consistent with this study’s conclusion [122]. Thus, metformin can serve as an anti-cancer drug for EZH2-overexpressing solid tumors in the future. In addition, another study reported that SMYD2-mediated EZH2-K307 di-methylation protects EZH2 from ubiquitination degradation. They also illustrated that SMYD2 together with EZH2 promotes the oncogenesis and metastasis of breast cancer cells, suggesting that SMYD2 inhibitor may become a novel underlying target for anti-breast cancer treatment [39].

## Conclusions and future directions

EZH2, as an important component of PRC2, is a critical player in the epigenetic regulation of gene expression. The last 25 years’ comprehensive study on EZH2 demonstrates a splendid landscape that a histone methyltransferase is able to participate in numerous biological processes, especially in oncogenesis and cancer distant metastasis. Meanwhile, EZH2 expression is also regulated by a lot of ways, among which post-translational modification of EZH2 occupies a very important position. So far, abundant data about various modification residues, modification types, and interacting enzymes have been shown, which improved understanding about EZH2 functions, mechanisms, regulations, and therapeutic applications. However, several issues remain to be discussed.

First, some modifying enzymes and precise residues of EZH2-PTMs remain largely unknown. Second, whether other types of PTMs, such as crotonylation, succinylation, and malonylation, also exist in EZH2 has yet to be determined. Moreover, EZH2 modification is a highly dynamic and context-dependent process, and crosstalks among modifications enlarge the functional spectrum of EZH2-PTMs. However, how these crosstalks of EZH2-PTMs are precisely regulated in cancer progression has yet to be elucidated.

A series of studies demonstrated that EZH2 can promote cancer tumorigenesis and metastasis independent on PRC2-mediated target gene silencing. Whether EZH2 has other important novel functions warrants further investigations. In future studies about EZH2-PTMs, these new roles of EZH2 should be considered. Several of the reports about EZH2-PTMs experiments were performed in vitro. These findings dependent on experiments in vitro could not be completely confirmed by the following experiments in vivo according to the past studies. These results in vitro should be validated by experiments in vivo in the future. This may explain why there is not any successful clinical trial targeting EZH2-PTMs for anti-cancer treatment to be carried out until now. Therefore, further investigations are still needed before the clinical application of anti-EZH2 PTMs in cancer therapy.

In conclusion, understanding the regulation of EZH2-PTMs and their crosstalks in cancer progression and demonstrating their molecular mechanisms in depth will open a promising way for the development of novel cancer therapeutic strategies.

## Data Availability

Not applicable.

## Abbreviations

EZH2: Enhancer of zeste homolog 2
PTMs: Post-translational modifications
HMTase: Histone methyltransferase
PRC2: Polycomb Repressive Complex 2
H3K27me3: Trimethylation of lysine 27 of histone H3
ER: Estrogen receptor
GBM: Glioblastoma multiforme
GlcNAc: *O*-GlcNAcylation
OGT: *O*-Linked *N*-acetylglucosamine transferase
HATs: Histone acetyltransferases
HDACs: Histone deacetylases
LSD1: Lysine-specific demethylase 1
PCAF: P300/CBP-associated factor
SMYD2: SET and MYND domain containing 2
Ub: Ubiquitin
Smurf2: Smad ubiquitination regulatory factor-2
c-Cbl: Casitas B-lineage lymphoma
CHIP: COOH terminus of Hsp70-interacting protein)
SUMO: Small ubiquitin-like modifier
DUBs: Deubiquitinases
USP7: Ubiquitin-specific protease 7
lncRNAs: Long noncoding RNAs
ncRBD1: NcRNA-binding domain
ADMA: Asymmetric di-methylation
TAMs: Tumor-associated maceophages
HCC: Hepatocellular carcinoma
NKTL: Natural killer/T-cell lymphoma
HGSOC: High-grade serous ovarian carcinoma
NIPP1: Nuclear inhibitor of PP1
YC-1: 3-(5′-Hydroxymethyl-2′-furyl)-1-benzylindazole
NEK2: NIMA-related kinase 2
GNA: Gambogenic acid
